# Specific Salivary Neuropeptides Shift Synchronously during Acute Stress in Fire Recruits

**DOI:** 10.3390/brainsci14050492

**Published:** 2024-05-13

**Authors:** Rebecca Ryznar, Nathan Andrews, Kyle Emery, Michaela Snow, Mark Payton, Francina Towne, Dean Gubler

**Affiliations:** 1 Department of Biomedical Sciences, Rocky Vista University, Centennial, CO 80112, USA; mpayton@rvu.edu (M.P.);; 2 College of Osteopathic Medicine, Rocky Vista University, Centennial, CO 80112, USA; nathan.andrews@co.rvu.edu (N.A.); kyle.emery@co.rvu.edu (K.E.); michaela.snow@co.rvu.edu (M.S.); 3 Department of Military Medicine, Rocky Vista University, Ivins, UT 84738, USA; dgubler@rvu.edu

**Keywords:** α-MSH, β-Endorphin, neuroimmune axis, neurotensin, oxytocin, Substance P

## Abstract

Once thought of as an immune-privileged site, we now know that the nervous system communicates in a bidirectional manner with the immune system via the neuroimmune axis. Neuropeptides constitute a component of this axis, playing critical roles in the brain and periphery. The function of salivary neuropeptides in the acute stress response is not well understood. The purpose of this study is to investigate salivary neuropeptide levels during acute stress. Salivary samples were collected from fire recruits engaged in a stress training exercise previously shown to induce acute stress, at three separate timepoints during the exercise and levels of oxytocin, neurotensin, Substance P, α-MSH, and β-Endorphin were measured using the Human Neuropeptide 5-Plex Custom Assay Eve Technologies. All neuropeptides increased throughout the acute stress simulation and during the recovery phase. Exploratory factor analysis (EFA) identified one factor contributing to baseline values across five neuropeptides and Pairwise Pearson Correlation Coefficient analysis showed positive correlations >0.9 for almost all neuropeptide combinations at the pre-stress timepoint. Further analysis identified negative and positive correlations between past-life trauma and self-assessed hardiness, respectively. Calculated neuropeptide scores showed an overall positive correlation to self-assessed hardiness. Altogether, our results suggest that salivary neuropeptides increase synchronously during acute stress and higher levels correlate with an increase in self-assessed hardiness. Further study is required to determine if interventions designed to enhance neuropeptide activity can increase stress resilience, especially in high-stress occupations such as firefighting.

## 1. Introduction

Stress is unavoidable. Whether physical or psychological, acute or chronic, mild or intensely traumatic, stress has the potential to compromise an individual’s health. Traumatic stress is associated with the poorest health outcomes [1]. It is estimated that 60% of women and 50% of men experience at least one trauma throughout their lives [1]. Some individuals display a resilient response to stress, while others develop PTSD (post-traumatic stress disorder). For healthcare providers such as trauma surgeons, emergency medical personnel, and police, rates of PTSD approach 11–15%, compared to 6% in the general population [2]. It is critical to investigate the underlying mechanisms for building stress resilience in these populations.

Stress occurs when a change inflicts physical or psychological strain and pressure upon individuals. Stress is highly variable and unique to the individual, by the nature of its origin, whether it is physical or psychosocial, its length of exposure, and its intensity. The variability in stress responses can be due to physiological and psychological differences such as the individual’s genetics, previous life trauma, and the neuroinflammatory state. When an individual is stressed, the SNS (Sympathetic Nervous System) is activated. The SNS is responsible for initiating the quick fight-or-flight response. The amygdala plays a crucial role in processing fear, arousal, and emotional stimuli to assess the appropriate response. When needed, the amygdala transmits a stress signal to the hypothalamus, which in turn triggers the activation of the sympathetic nervous system (SNS). Consequently, the adrenal glands release a surge of catecholamines, including epinephrine. As the body persists in perceiving the stimuli as a threat, the hypothalamus initiates the hypothalamic–pituitary–adrenal (HPA) axis, leading to the release of cortisol from the adrenal cortex. The secretion of glucocorticoids provides a negative feedback loop for inhibiting the release of Corticotropin-Releasing Hormones (CRHs) and adrenocorticotropic hormones (ACTHs) from the hypothalamus and anterior pituitary, respectively [3]. Additional critical regulators of the stress response include neuropeptides and catecholamines, with both acting as neurotransmitters within circuits of the central and peripheral nervous systems mediating both systemic and psychological stress responses. Neuropeptides and catecholamines have been implicated in regulating long-term adaptation and maladaptation to stress as measures of activity or lack of activity and have been found to correlate with stress-resilience states [4].

Neuropeptides represent the largest and most diverse class of signaling molecules in the nervous system [5]. Typically binding to G protein-coupled receptors (GPCRs) of cells within the neuroendocrine axis, neuropeptides can modulate immune, neural activity, and other tissues like the gut, muscles, and heart [5]. Neuropeptides, often released alongside other neuropeptides and neurotransmitters, possess the ability to diffuse extensively, impacting many targets. Unlike classical neurotransmitters like acetylcholine or epinephrine, neuropeptides act slowly and exert prolonged effects, contrasting with the rapid and short-term responses of the neurotransmitter [6]. Despite their presence in tissues at significantly lower concentrations compared to classical neurotransmitters, neuropeptides remain active at receptors, even at these reduced levels [6]. Moreover, neuropeptides are not only released from nerve endings but also from inflammatory immune cells such as monocytes, dendritic cells, eosinophils, and mast cells [6]. Functional roles of neuropeptides remain relatively overlooked or described as modulatory, but numerous studies suggest they are involved in central regulation and mediation of numerous key systemic processes, including the stress response [7,8,9,10,11,12,13]. Neuropeptides have been found to be important for the adaptation of the nervous system to changes in physiological conditions, potentially regulating resilience to stress and protection against stress-related disorders such as PTSD [7]. Numerous neuropeptides, such as Substance P, Oxytocin, Neurotensin, and Neuropeptide Y (NPY), have been shown to be affected by stress or to be involved in stress response in various animal models and human studies [7,8,9,10,11,12,13,14,15,16,17].

Specific neuropeptides released from the CNS following stress can communicate with immune cells in the periphery [17]. This communication occurs anywhere in the body where nerve fibers releasing neuropeptides encounter cells of the immune system or where bloodborne neuroendocrine-immune mediators meet with nerve fibers [15] or by which immune cells produce neuropeptides that can feed back to the CNS [5,6,7]. Since neuropeptides not only modulate the stress response but can serve as central regulators, it has become a viable possibility to utilize these peptides as potential targets for the detection of stress susceptibility but also for diagnosis and treatment of stress-related breakdowns. Neuropeptide measurements can deepen our understanding of the pathophysiology of the stress response, something self-reports cannot capture. The detection of stress-induced neuropeptide changes could lead to better diagnostic tools and treatments. If certain NPs are found to be predictive of better stress resilience, interventions could be created to enhance NP activity. Quantitative levels of NPs could also provide data points that could be tracked over time in response to interventions. Neuropeptides have indeed been targeted for drug development or use; for example, Neuropeptide Y for anxiety [8] and intransal oxytocin for PTSD [9]. Many studies report findings of neuropeptides, such as oxyotcin and neurotensin, correlating with resilience and attenuating depression, anxiety, PTSD, suicide, and other stress-related behavior [10,11,12,13,14,15,16,17]. 

Research conducted on neuropeptides in humans has been studied by sampling regions of the CNS, cerebrospinal fluid (CSF), or blood, but little is known about how salivary levels of neuropeptides function in relation to stress responses and stress resilience. The salivary glands are innervated [18] and suggested as a possible route for peptide access to saliva. Therefore, neuropeptides may be released from nerve terminals in and around the salivary glands [19]. It is possible that salivary levels of neuropeptides may reflect the collective tone of the nervous system as proposed by Kallman et al., whereby changes in basal activity in the nervous system would be reflected in altered salivary levels [20]. Investigating neuropeptide changes and combining this information with self-reports can provide a more comprehensive insight into stress and coping, which could lead to a multi-pronged approach to treatment. Due to the nature of their high-stress work environment and the need to investigate potential mechanisms for reducing PTSD, our research setting involved firefighters. The aim of the present study was to investigate salivary neuropeptide concentrations during acute stress and determine if there is a correlation between salivary neuropeptide levels, an individual’s experienced life trauma, and perceived stress resilience.

## 2. Materials and Methods

### 2.1. Study Design

This is a prospective cohort study investigating levels of salivary neuropeptides of participants at baseline, during, and after a psychophysical challenge with both emotionally and physically stressful components. Rocky Vista University College of Osteopathic Medicine approved the study through its IRB committee, which included extension and modification from a previous study (IRB#2019-0092) [21]. The study was designed and conducted in accordance with the Declaration of Helsinki. All participants were consented, with first responders enrolled in the fire academy training program at a local fire department in the state of Colorado. In addition to completing a demographics survey (Supplemental Table S1) and providing salivary samples, participants also completed the Life Events Checklist (LEC-5) questionnaire [22] to assess life trauma and the Hardiness Resilience Gauge (HRG) questionnaire [23] to assess resilience post-completion of the training.

The study was performed from June to July of 2022. Three sets of saliva samples were gathered for analysis. Collection occurred prior to the stressful event, immediately after, and one hour post-event. Participants were selected based on their enrollment in the fire academy. Saliva collection started with pre-stress samples taken before the event, with the first recruit starting at 8:00 am. The stress event occurred within two hours of acquiring pre-stress samples. Post-stress samples were collected immediately after the event’s conclusion. Recovery samples were obtained one hour post-event. Saliva collection utilized the whole stimulated saliva method as outlined by Ryznar et al. [21]. This involved participants chewing sugar-free gum for five minutes, followed by the collection of 1 mL of saliva in 1.5 mL eppendorf tubes. Upon collection, a protease inhibitor solution with a concentration of 1 μg/mL was added, and the samples were stored on ice. The samples were shipped on dry ice and concentrations were determined using the bead-based Human Neuropeptide 5-Plex Neuropeptide assay from Eve Technologies (Calgary, AB, Canada). Luminex xMAP technology for multiplexed quantification of five human neuropeptides (oxytocin, neurotensin, α-MSH, β-endorphin, and Substance P).

The multiplexing analysis was performed using the Luminex™ 200 system (Luminex, Austin, TX, USA) by Eve Technologies Corp. (Calgary, AB, Canada). Five markers were simultaneously measured in the samples using Eve Technologies’ Human Neuropeptide 5-Plex Custom Assay (MilliporeSigma, Burlington, MA, USA) according to the manufacturer’s protocol. The five-plex consisted of α-MSH, β-Endorphin, neurotensin, oxytocin, and Substance P. Assay sensitivities of these markers range from 10–85 pg/mL for the five-plex. Individual analyte sensitivity values are available in the MilliporeSigma MILLIPLEX^®^ MAP protocol. Neuropeptides were reported in units of pg/μL. All samples were shipped on dry ice from Colorado to Eve Technologies (Calgary, AB, Canada). 

### 2.2. Participant Population and Stress Test

To complete the fire academy training, all fire recruits were required to pass a physical and psychological stress test as previously described in Ryznar et al. [21]. As shown in Ryznar et al., this standardized test does in fact illicit an acute stress response [21]. Briefly, the participants were subjected to a challenging scenario wherein they were blindfolded, distracted, and tasked with navigating through a simulated collapsing building while ensuring that their air masks remained on. Success in keeping the air mask on was considered a passing grade for the test. This combination of physical and emotional stress aimed to prepare fire recruits for the rigorous demands of training in real-life burning buildings and the perpetual high-stress environment of the job. The test was meticulously designed to push participants to their stress limits within the safety of a simulated setting. In an actual fire situation, the consequences of removing the mask could be fatal, as even a brief exposure to super-heated and toxic smoke could render individuals unconscious.

### 2.3. Hardiness Resilience Gauge and Life Events Checklist

After each participant completed the HRG questionnaire, scores were provided through a technology called Mental Health Systems (MHS). Total scores were determined by adding the three dimensions of hardiness together: control, commitment, and challenge. The Life Events Checklist (LEC-5) is a self-reported measure of past trauma. There are 17 questions scored from 1–6, with 1 being least traumatic and 6 being most traumatic. It is used to reliably aid in the diagnosis of PTSD. After completion of the LEC-5 questionnaire, a severity score was assigned as described in Speakman et al. [24]. Briefly, the score was assigned based on the number of traumatic events experienced (assigned a score of 3 for each), witnessed (assigned a score of 2), and learned about (assigned a score of 1).

### 2.4. Statistical Analysis

Data collection resulted in salivary concentrations for five neuropeptides (Substance P, oxytocin, neurotensin, ß-Endorphin, and α-MSH) across 24 participants. All reported concentrations were utilized for analysis with values in excess of the standard curve reduced to the highest observed concentration within the standard curve.

Statistical methods, including Pearson correlation coefficients and exploratory factor analyses, were then utilized to extract the relationships between five different neuropeptides collected at the following time points: pre-stress (t1), post-stress (t2), and 1 h post-stress (t3). Descriptive statistics for each neuropeptide and time point are shown in Supplemental Material and were found to be normally distributed. Due to the variability of our dataset at pre-stress for all five neuropeptides, a relative percent change was calculated and utilized to represent neuropeptide fluctuation throughout the acute stress simulation and recovery time periods, PreVPo (pre versus post timepoints) and PoVRe (post versus recovery timepoints), respectively. Individual participant neuropeptide trends, as well as aggregates, were identified and quantified to assess the ability of the acute stress simulation to elicit a measurable acute stress response.

In order to evaluate a common neural pathway, the linear relationships between individual neuropeptides were evaluated using Pearson Correlation Coefficients (r-values) for all combinations of neuropeptides at three timepoints: pre-stress, PreVPo, and PoVRe. Coefficients were then categorized to quantify the strength of the relationship. 

Analysis was further conducted to evaluate how past life trauma and self-perceived hardiness, LEC-5, and HRG scores, respectively, correspond to the five neuropeptide levels throughout acute stress simulation. Pearson Correlation Coefficients were generated for each individual neuropeptide compared to both LEC-5 and HRG at each timepoint: pre-stress, post-stress, recovery, PreVPo, and PoVRe. Coefficients were then categorized to quantify the strength of the relationship.

Data analysis was conducted using R-studio Version 2022.12.0+353 [25] for the creation of a neuropeptide score. This value represented total neuropeptide movement throughout the acute stress simulation. Exploratory factor analysis was utilized and performed on the participants’ neuropeptide data. This approach aims to reduce dimensionality and explain the observed correlation among variables by identifying underlying factors. Further analysis can then be simplified using these factors [26]. Eigenvalues were generated and used to explain the amount of variance explained by each factor [27]. Using the Kaiser Criterion, only factors with an Eigenvalue greater than one were included [24]. Loadings less than 0.4 were then suppressed following the suggestion of Stevens who claims the cutoff, irrespective of sample size, for interpretive purposes [28]. Data confidentiality: consent forms included information about data storage and confidentiality. All data were stored only until analysis was completed. No results were identifiable other than the consent forms linking the participant to a randomized four-digit code.

For the creation of a neuropeptide score, regression-based factor scores were generated with the aim of predicting the location of individuals within each factor. The factor scores are then standardized to a mean of zero [29]. In order to represent all five neuropeptides at each time point, these factor scores were used to represent individuals across the four factors.

As the neuropeptide score represents the movement of all five neuropeptides in an acute stress simulation, it can be utilized to evaluate a potential neuronal axis. In a similar manner as before to evaluate the effect of past life trauma and self-perceived hardiness, Pearson Correlation Coefficients were generated between each neuropeptide score and their Lec5 and Post-HRG scores.

## 3. Results

A total of 24 participants comprised the study cohort. All participants completed a demographics survey displayed in Supplemental Table S1. This survey included information about sex, gender, age, heritage, emergency medical training, military exposure, and previous post-traumatic stress disorder diagnosis. Of the 24 participants, 22 (91.6%) were male with a mean age of the cohort 28.8 +/− 3.8 years. Twenty-one (87.5%) participants reported prior experience as an emergency medical technician (EMT), paramedic, firefighter training, and/or military. Five (20.8%) participants specified current or prior psychiatric diagnoses, including depression, anxiety, and PTSD. Of 24 recruits, 79% were Caucasian, 8.3% Black, 4.1% were Asian American, and 4.1% were mixed race.

### 3.1. Neuropeptide Fluctuations throughout Acute Stress and Recovery

**Figure 1:**
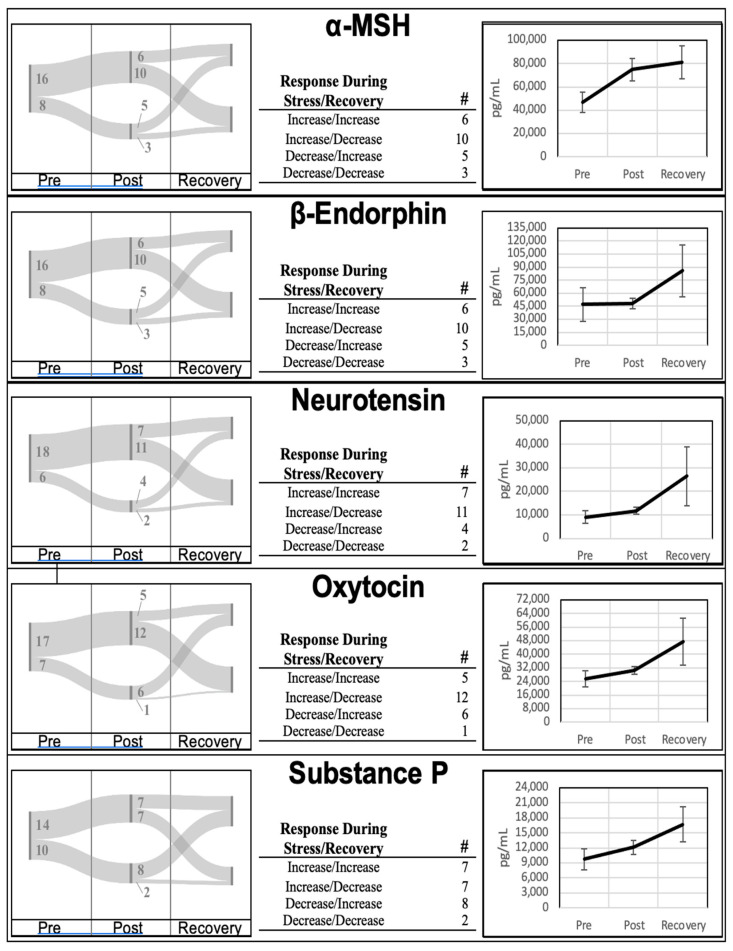
Neuropeptide movement is highly individualized. Neuropeptide aggregates increase during acute stress events and recovery. Time points are pre-stress (immediately before stress test), post-Stress (immediately following stress test), and recovery (1 h after stress test). All neuropeptides were measured using bead-based multiplex Elisa from Eve technologies. Neuropeptide concentrations are in pg/mL. Representative error bars are ± SEM (standard error of the mean). Sample sizes n = 24.

Neuropeptide levels from all participants were collected at the three timepoints (pre, post, and recovery). Mean neuropeptide concentrations at the pre-stress, post-stress, and recovery time points are highlighted in Figure 1. Aggregates of all five neuropeptides collected in this study increased during the acute stress encounter (PreVPo) and continued to increase during the recovery phase (PoVRe). Descriptive statistics for each time point and neuropeptide are found in Supplemental Table S2.

The aggregate trend of increasing at both PreVPo and PoVRe is only followed by 20%-to-30% of the participants over all five neuropeptides. A review of individual trends for each neuropeptide is displayed in Figure 1. For all neuropeptides except Substance P, 40%-to-50% of the participants displayed an increase in concentration at PreVPo and a decrease at PoVRe. The remaining 20%-to-30% of participants displayed a decrease at PreVPo for these four neuropeptides. Substance P displayed a more even distribution across the possible neuropeptide movement with roughly 31% of participants representing the three major trends: an increase at both PreVPo and PoVRe, an increase at PreVPo with decrease at PoVRe, and a decrease at PreVPo and increase at PoVRe. Up to 13% of participants were presented with decreased levels of all neuropeptides at PreVPo and PoVRe.

### 3.2. Neuropeptide Relationships

**Figure 2:**
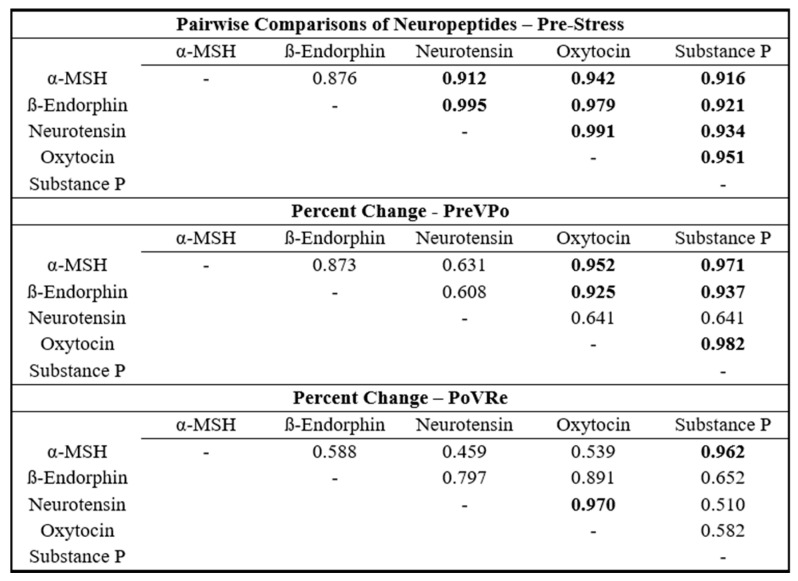
Pair-wise Pearson Correlation Coefficient Matrix for the five neuropeptides throughout the acute stress response. Time periods are: Pre-stress, percent change during acute stress (PreVPo), and percent change during a 1 h Recovery (PoVRe). Correlation coefficients greater than 0.9 have been bolded.

To assess the interrelationships of the neuropeptides, Pearson correlation coefficient matrices were generated for each combination of neuropeptides at the three time points: pre-stress, PreVPo, and PoVRe. The results are shown in Figure 2. Assessing combinations of neuropeptides at pre-stress displayed Pearson correlation coefficients of no less than r = 0.880. PreVPo displayed a greater range between r = 0.608 and r = 0.982 and PoVRe displayed a range between r = 0.459 and r = 0.970.

### 3.3. Neuropeptide Score, LEC-5, and Post-HRG Profile Comparison

**Figure 3:**
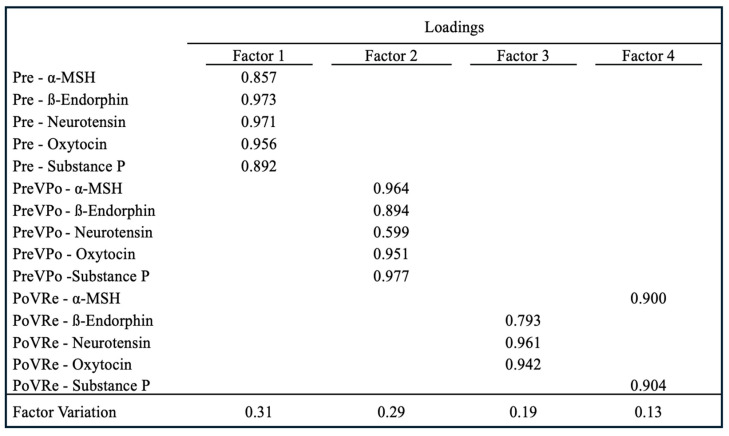
Factor analysis loadings for the five neuropeptides throughout the acute stress response. Time periods are: Pre-stress, percent change during acute stress (PreVPo), and percent change during a 1 h recovery (PoVRe). Only loadings ≥0.4 have been kept based on the suggestion of Stevens [28].

To determine a potential common neural axis, a neuropeptide score was calculated to identify and quantify all neuropeptide values at each timepoint. As shown in Figure 3, EFA resulted in four factors representing 31%, 29%, 19%, and 13% variation, respectively. The *p*-value for the EFA is *p* < 0.01. All five neuropeptides at pre-stress loaded on Factor 1 with loadings ranging from 0.857 to 0.973. At PreVPo, all neuropeptides loaded on Factor 2, with loadings ranging from 0.599 to 0.977. ß-Endorphin, neurotensin, and oxytocin loaded on Factor 3 at PoVRe, with loadings ranging from 0.793 to 0.961, while α-MSH and Substance P loaded on Factor 4 at PoVRe, with loadings ranging from 0.900 to 0.904.

**Figure 4:**
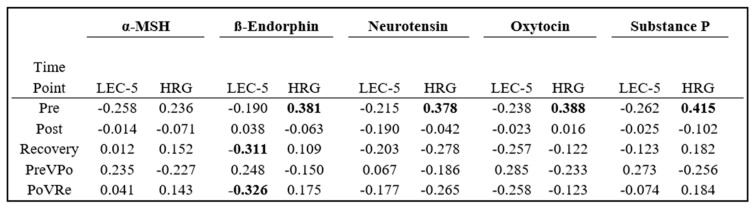
Pearson Correlation Coefficients of neuropeptides and LEC-5 or HRG scores. Values ≥ ±0.3 are presented in bold. Time periods are: pre-stress, post-stress, 1 h post-stress, percent change during stress (PreVPo), and percent change during recovery (PoVRe).

Analysis was conducted to evaluate how previous life trauma and self-perceived hardiness related to an individual’s neuropeptide levels. Pearson Correlation Coefficients were generated using participants’ LEC-5 and HRG scores for the three time points, as well as at PreVPo and PoVRe. These results are displayed in Figure 4. 

At pre-stress, all five neuropeptides displayed negative correlations, with LEC-5 scores ranging between −0.190 and −0.262 and positive correlations with HRG scores ranging from 0.236 and 0.415. ß-Endorphin displayed negative correlations negative correlations with LEC-5 scores at both the recovery and PoVRe time points. All other neuropeptides at the remaining time points displayed correlations between −0.3 and 0.3.

Each participant’s neuropeptide score for each factor was correlated to both its LEC-5 and HRG scores. An analysis with LEC-5 displayed neuropeptide scores from Factor 1 (reflective of pre-stress) and Factor 3 (reflective of ß-Endorphin, neurotensin, and oxytocin at PoVRe) as negative correlations, while Factor 2 (reflective of PreVPo) and Factor 4 (reflective of α-MSH and Substance P at PoVRe) are presented with positive correlations. HRG analysis presented differently, with Factor 1 and Factor 4 displaying positive correlations, while Factor 2 and Factor 3 displayed negative correlations. 

## 4. Discussion

Neuropeptides have been shown to be critical for regulating stress responses [8,9,10,11,12,13,14,15]. While some neuropeptides function to reduce stress, others can increase the stress response. Oxytocin, α-MSH, and β-endorphin seem to share this attenuating effect on stress response through the HPA axis. OT has been shown to release in response to stress and attenuate stress-induced responses, such as cortisol release [30]. α-MSH has been shown to inhibit stress-induced feeding behavior and reduce anxiety-like behaviors [31]. β-Endorphins are part of the endogenous opioid system and can reduce pain, inhibit an exaggerated immune response, and reduce stress [32].

On the other hand, Substance P and neurotensin activate the HPA axis in response to pain and stress. Substance P primarily acts at the level of the hypothalamus through stimulating the release of corticotropin-releasing hormone (CRH) [33]. Neurotensin also activates the HPA axis in response to pain and stress, but its actions are two-fold: stimulating the release of both CRH from the hypothalamus and ACTH from the pituitary gland [34]. The activity of both neuropeptides leads to a release of cortisol, an activation of the SANS, and the preparedness of the body to handle stress through the mobilization of energy sources and heightened arousal [33,34].

With stress-related disorders on the rise, it is critical to investigate potential biomarkers of stress resilience and stress recovery for those who work in trauma healthcare. While much is known about neuropeptide functions in the CNS [35,36], salivary neuropeptide functions and their relation to stress are not as well understood but are an invaluable methodology for clinical diagnostics [18]. Our study investigated levels of salivary neuropeptides during acute stress and how these relate to life trauma and resilience scores. Investigating neuropeptide changes and combining this information with self-reports can provide a more comprehensive insight into stress and coping, which could lead to a multi-pronged approach to treatment. Using a setting previously shown to induce acute stress as evidenced by an increase in known inflammatory markers of acute stress [21,24], we report highly variable shifts in neuropeptide concentrations across our cohort (Figure 1). Even though there was great variability in how these neuropeptides shifted during acute stress across our cohort, the Pearson correlation coefficient analysis revealed strong correlations between neuropeptides at various time points, suggesting salivary neuropeptides may shift synchronously in individuals throughout acute stress.

Our results show the strongest and most consistent positive correlations at a timepoint immediately prior to stress (time point 1 or pre-stress). At this time point, almost all neuropeptides approach an r value of close to 1. For the percent change immediately after stress and during the recovery period (1 h after), some salivary neuropeptide correlations are uncoupled. Neurotensin was unique, as it did not show a strong correlation during the pre-stress period. A strong association was found during the post to recovery phase, especially with oxytocin (r value of 0.9695 Figure 2). Previous reports suggest neurotensin is responsible for assigning valence (positive or negative emotion) to a stressful experience [37]. It is suggested that the brain’s default response is to have a bias toward fear, and neurons associated with negative valence are activated until neurotensin is released. Neurotensin activates neurons associated with positive valence [37]. It is not surprising to see oxytocin levels correlating with neurotensin during recovery as both neurotensin and oxytocin release are associated with positive emotions [37,38,39]). Strong salivary neuropeptide correlations persisting throughout the acute stress response were seen with oxytocin: α-MSH/ß-Endorphin/Substance P and Substance P: α-MSH/ß-Endorphin/oxytocin. Additionally, during recovery, Substance P and α-MSH showed a strong correlation (r value of 0.9622). Both neuropeptides have been reported as anxiogenic and their blockade can reduce anxiety [20,40]. Also, a previous study supports our finding of a strong positive correlation between Substance P and ß-Endorphin [41]. Our exploratory factor analysis evaluated relationships between our neuropeptides during acute stress by identifying factors contributing to variation across our cohort. The analysis revealed that salivary neuropeptide levels at pre-stress (T1) constitute the latent variable of Factor 1, suggesting levels prior to stress may be predictive for variation from stress to recovery (Figure 3).

Based on numerous reports of neuropeptides modulating the stress response and possibly serving protective functions against the negative effects of traumatic stress [42,43], such as PTSD, we were curious to determine if salivary levels had any association with an individual’s trauma experiences or resilience to stress. The one salivary neuropeptide we found moderately correlating with high LEC-5 scores or significant trauma backgrounds was ß-Endorphin concentrations during the recovery period. Greater increases in the levels of ß-Endorphin in saliva from stress to recovery seem to be associated with lower LEC-5 scores, or less life trauma experienced by the participant (Figure 3). Previous studies have shown results from the brain tissue of PTSD-like and non-PTSD-like rats found significantly lowered levels of β-endorphin in the amygdala of the PTSD-like group [43], although post-exercise plasma beta-endorphin levels have been found to be significantly higher than resting levels in the PTSD patients only, suggesting a more exaggerated response [43]. Furthermore, one study has shown that an attenuated beta-endorphin response to acute stress is associated with smoking relapse [44]. Therefore, it is critical to further investigate the relationship between central and peripheral levels of neuropeptides during various stressors.

Our results show moderate positive correlations between all neuropeptides, other than α-MSH at pre-stress (T1), and resilience scores using the HRG. Several studies suggest that the activation of the α-MSH receptor is anxiogenic, while the blockade has been shown to reduce anxiety [45]. Other studies have shown associations between salivary levels of ß-Endorphin, oxytocin, Substance P, and stress resilience [39,40,43]. One study showed that Substance P can act as an inhibitory molecule for ACTH and glucocorticoids under basal conditions [46]. No study to our knowledge has reported salivary levels of neurotensin associated with stress resilience in humans. Research has demonstrated that oxytocin is linked to heightened resilience in the aftermath of prolonged stress or adversity. In female students with high basal salivary oxytocin levels, a positive affect, and enhanced cognitive accuracy after stress (an emotionally stressful video) were observed, suggesting oxytocin may buffer the negative emotional effects of stress and can facilitate resilience [47]. Overall, many studies have supported the notion that neuropeptides are protective during stress and our results support these findings.

**Figure 5:**
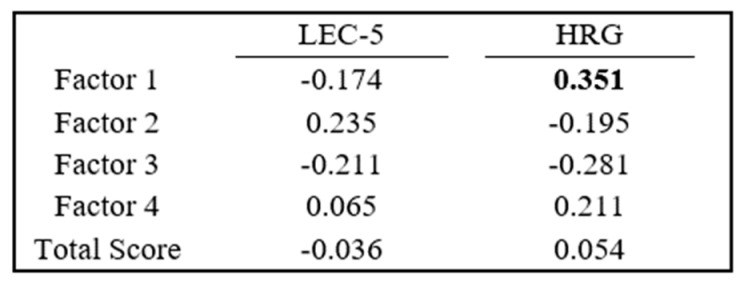
Pearson Correlation Coefficients for the neuropeptide scores compared to LEC-5 and HRG scores. Correlation coefficients greater than 0.3 have been bolded.

Since we saw strong positive correlations (approx. = 1) for our salivary neuropeptides measured during acute stress, we constructed an overall neuropeptide score to reflect the movement of all neuropeptides. Our neuropeptide score results showed a moderate correlation with resilience scores as assessed by the HRG (Figure 5). This correlation was with Factor 1 neuropeptide scores and high HRG scores, suggesting high basal levels of salivary neuropeptides may be predictive for individuals who are less stress reactive and may have healthier stress responses. Worth noting, results from an additional study using the HRG, where researchers found that lower neuropeptide Y levels in blood were associated with participants who were more stress reactive, suggested a high HRG score is linked to healthier neuroendocrine responses to stress [48], a finding similar to the observation in our study.

Our study has many limitations. Our small and rather homogenous population of mostly biological male fire recruits warrants additional studies with a larger, more diverse sample. Additionally, the non-standardization of the flow rate, along with the stimulated saliva-collection method, could introduce potential error through changes in pH that may skew our salivary results. It is unknown whether the salivary levels of these neuropeptides are derived from ultrafiltration from plasma, direct local production in the salivary glands, or a combination of both. We did not measure individual perceived levels of stress from these recruits, although we have determined this training exercise does induce an acute stress response [21,24]. Future studies should determine how these salivary levels correlate with susceptibility to stress-related pathologies such as depression or PTSD. Also, it is worth noting that our three-sample sequence approach was designed to detect individualized stress responses with the initial sample serving as the negative control, but we did not have access to a positive control. A future investigation could consist of collecting samples from individuals with high resilience. Additionally, we do acknowledge that other published studies report salivary levels of our neuropeptides at lower concentrations (low pg/mL), although some research groups report concentrations within our measured levels of high pg-mg/mL levels [49,50,51]. A future study is needed to compare detected salivary levels using different protein methods and in a more diverse population. 

## 5. Conclusions

Altogether, research into these neuropeptides can enhance our understanding of the biological processes that regulate stress reactions and an individual’s capacity for coping with stressors related to trauma. While evaluating biomarkers for stress may provide new insights, we must weigh these against cost-effective and convenient psychometric tests already in use. Future use could be considered for combining psychometric tests with biological markers to align perceived stress with physiological data.

Additionally, lifestyle changes, such as meditation [52], social bonding [53], and exercise [54], have been shown to increase circulating neuropeptides, which may buffer the effects of trauma and stress in the general population. Altogether, our results suggest that salivary neuropeptides synchronously shift together during acute stress, reflecting an individual’s overall nervous system tone, and may be used as a predictor for individual stress resilience. This study provides valuable insight into possible potential pharmacological targets for stress-related illness in both high-stress occupations and the general population.

## Data Availability

The original contributions presented in the study are included in the article/supplementary material, further inquiries can be directed to the corresponding author. The data are not publicly available due to their containing information that could compromise the privacy of research participants.

## Supplementary Materials

The following supporting information can be downloaded at: https://www.mdpi.com/article/10.3390/brainsci14050492/s1, Table S1: Demographics Data; Table S2: Descriptive Statistics Data.
