# Supplementary material for: Specific Salivary Neuropeptides Shift Synchronously during Acute Stress in Fire Recruits
Source: Brain Sci. 2024 May 13;14(5):492. doi: 10.3390/brainsci14050492 (PMC11119501; doi:10.3390/brainsci14050492)
Supplement: Supplementary file 1 [file brainsci-14-00492-s001.zip › brainsci-2956125-supplementary.pdf]

Supplemental Tables

Supplemental Table S1—Demographics Data

	Number	%
Gender		
Male	22	91.7
Female	2	8.3
Age		
20-30	15	62.5
30-40	9	37.5
Race		
White	21	87.5
Black	2	8.3
Asian	1	4.2
Pacific Islander	0	0
Native American	0	0
Ethnicity = Hispanic	2	8.3
Prior Military Experience	3	12.5
Prior Dx of PTSD	2	8.3
	Mean(Std. Dev.)	Range
Prior EMT Experience	6.54	3-15

n=24

Supplemental Table S2—Descriptive Statistics data

Time Point 1

Variable	Mean	Std Dev	N	Median	Minimum	Maximum
α-MSH	46728.11	40950.24	24	40691.87	2312.13	200233.00
ß-Endorphin	47217.32	93332.21	24	28263.25	3878.74	486672.87
Neurotensin	9130.17	12287.15	24	6838.25	1393.87	66090.47
Oxytocin	25499.64	23225.90	24	22090.52	3999.24	129435.74
Substance P	9765.98	10029.94	24	7066.87	824.64	51221.40

Time Point 2

Variable	Mean	Std Dev	N	Median	Minimum	Maximum
α-MSH	74845.15	46459.35	24	60424.90	13206.48	186345.12
ß-Endorphin	48231.87	29307.81	24	40399.10	11975.40	116304.85

Neurotensin	11787.72	7176.59	24	10577.34	3489.04	39111.77
Oxytocin	30593.81	11299.19	24	28596.01	11395.82	51203.16
Substance P	12082.35	6513.47	24	10119.87	2778.03	26453.02

Time Point 3

Variable	Mean	Std Dev	N	Median	Minimum	Maximum
α-MSH	80833.44	67666.29	24	59833.63	2083.43	200233.00
ß-Endorphin	85903.59	142537.29	24	34621.97	3281.83	116304.85
Neurotensin	26507.88	60120.97	24	8910.79	808.46	300000.00
Oxytocin	47393.27	65679.87	24	29050.90	3306.36	317867.88
Substance P	16685.82	16544.47	24	10731.83	821.42	51221.40
